# Supplementary material for: Water-Soluble closo-Docecaborate-Containing Pteroyl Derivatives Targeting Folate Receptor-Positive Tumors for Boron Neutron Capture Therapy
Source: Cells. 2020 Jul 3;9(7):1615. doi: 10.3390/cells9071615 (PMC7407413; doi:10.3390/cells9071615)
Supplement: Supplementary file 1 [file cells-09-01615-s001.pdf]

Supporting Information

Water-soluble *closo*-Docecaborate-Containing Pteroyl Derivatives Targeting Folate Receptor-Positive Tumors for Boron Neutron Capture Therapy

Tomoko Nakagawa,[a] Hidehisa Kawashima,[b] Taiki Morita,[a,b] and Hiroyuki Nakamura*,[a,b]

[a] *School of Life Science and Technology, Tokyo Institute of Technology, 4259 Nagatsuta-cho, Midori-ku, Yokohama 226-8503, Japan.*
[b] *Laboratory for Chemistry and Life Science, Institute of Innovative Research, Tokyo Institute of Technology, 4259 Nagatsuta-cho, Midori-ku, Yokohama 226-8503, Japan.*

List of contents

Spectral Date of PBC 1-4 S2
Figure S1. Western blot analysis of FRα in various cell lines. S3
Figure S2. Quantitative analysis of folate receptor (FRα) expressed in cells by flow cytometry using anti-FRα antibody. S4
Figure S3. Time-dependent boron accumulation in HeLa cells. S5
Figure S4. The concentration-dependent cell viability of folate in HeLa cells. S5

Spectral Date of PBC 1-4

NMR spectra were recorded on a BRUKER BIOSPIN AVANCE II 400 (400 MHz for ^{1}H) or a BRUKER BIOSPIN AVANCE III HD500 (125 MHz for ^{13}C, 160 MHz for ^{11}B) in the indicated solvent. Chemical shifts are reported in units of parts per million (ppm) relative to tetramethylsilane (0.00 ppm), $CDCl_3$ (7.26 ppm), DMSO-d_6 (2.50 ppm), CD_3CN (1.94 ppm) or D_2O (4.79 ppm) for ^{1}H NMR and $CDCl_3$ (77.2 ppm), DMSO-d_6 (39.5 ppm) or CD_3CN (1.32 ppm) for ^{13}C NMR. Boron trifluoride diethyl ether complex ($BF_3{\cdot}OEt_2$) was used as the external standard for ^{11}B NMR spectra. Multiplicities are reported by using the following abbreviations: s; singlet, d; doublet, dd; doublet of doublets, t; triplet, tt; triplet of triplets, sext.; sextet, m; multiplet, *J*; coupling constants in Herts (Hz). IR spectrum was recorded on a JASCO Corporation FT/IR-4100 FT-IR Spectrometer. Only the strongest and/or structurally important peaks were reported as the IR data given in cm^{-1}. HRMS (ESI-TOF-MS) were measured with Bruker micrOTOF II.

PBC 1: a brown solid. ^{1}HNMR (400 MHz, D_2O) δ 8.66 (s, 1H), 7.64 (d, *J* = 8.5 MHz, 2H), 6.77 (d, *J* = 8.5 MHz, 2H), 4.59 (s, 2H) , 3.66-3.71 (m, 8H), 3.56 (t, 2H); ^{13}CNMR (125 MHz, D_2O) δ 170.4, 150.8, 149.8, 148.4, 135.7, 129.1, 127.7, 121.9, 112.6, 71.0, 68.8, 67.6, 45.8, 39.5; ^{11}BNMR (160 MHz, D_2O) δ 6.36, -16.40, -18.28, -23.33; HRMS (ESI, negative): m/z calcd. for $C_{18}H_{31}B_{12}N_7O_4Na$ $[M+Na]^-$: 562.3541, found 562.3503; IR (KBr): 3566, 3391, 2928, 2871, 2482, 2361, 1605, 1510, 1304, 1176, 1159, 1118, 1057, 1029, 943, 837, 822, 768 cm^{-1}; mp > 300 °C.

PBC 2: a brown solid. ^{1}HNMR (400 MHz, D_2O) δ 8.70 (s, 1H), 7.69 (d, *J* = 8.3 MHz, 2H), 6.85 (d, *J* = 8.2 MHz, 2H), 4.65 (s, 2H) , 4.32 (t, 1H), 3.54-3.31 (m, 6H), 3.13-3.17 (m, 4H), 2.56-2.10 (m, 4H); ^{13}C-NMR (125 MHz, D_2O) δ 178.8, 175.7, 169.2, 167.3, 157.9, 153.6, 150.8, 148.5, 148.3, 129.2, 129.0, 129.0, 127.4, 121.2, 112.4, 70.9, 68.3, 67.5, 55.3, 45.6, 32.7, 27.7; ^{11}BNMR (160 MHz, D_2O) δ 6.16, -16.33, -18.22, -22.9; HRMS (ESI, negative): m/z calcd. for $C_{23}H_{38}B_{12}N_8O_7Na$ $[M+Na]^-$: 691.3970, found 691.3979; IR (KBr): 3567, 3385, 2926, 2856, 2733, 2485, 2361, 1682, 1606, 1508, 1304, 1176, 1159, 1109, 1065, 1026, 951, 839, 822, 770, 723 cm^{-1}; mp > 300 °C.

PBC 3: a brown solid. ^{1}HNMR(400 MHz, DMSO-d_6) δ 8.47 (s, 1H), 7.51 (s, 2H), 6.61 (s, 2H), 4.37 (s, 2H), 3.58-3.45 (m, 6H), 3.35-3.27 (m, 4H), 2.64 (s, 2H); ^{13}CNMR (125 MHz, DMSO-d_6) δ 173.8, 172.0, 169.8, 154.2, 151.2, 146.9, 129.2, 112.4, 71.0, 70.9, 68.6, 68.5, 67.6, 55.3, 48.9, 45.5, 43.1, 39.1, 38.7; ^{11}BNMR (160 MHz, DMSO-d_6) δ 6.12, -16.79, -17.98, -22.95; HRMS (ESI, negative): m/z calcd. for $C_{20}H_{34}B_{12}N_8O_5Na$

$[M+Na]^-$: 619.3757, found 619.3711; IR (KBr): 3568, 3368, 2931, 2873, 2491, 2371, 1605, 1508, 1445, 1307, 1182, 1114, 1069, 943, 836, 822, 769 cm^{-1}; mp > 300 °C.

PBC 4: a brown solid. ^{1}HNMR(400 MHz, DMSO-d_6) δ 8.63 (s, 1H), 7.65 (d, J = 7.9 MHz, 2H), 7.68 (d, J = 8.0 MHz, 2H), 4.60 (s, 2H), 3.55-3.77 (m, 12H), 3.32 (t, 1H), 3.22-3.17 (m, 3H); ^{13}CNMR (125 MHz, D_2O) δ 178.0, 175.9, 170.0, 160.5, 148.3, 150.6, 130.9, 129.2, 127.8, 112.5, 71.5, 68.8, 68.4, 67.6, 55.2, 50.6, 49.7, 45.8, 39.1, 38.7; ^{11}BNMR (160 MHz, D_2O) δ 5.84, -16.58, -18.45, -23.7, HRMS (ESI, negative): m/z $[M+Na]^-$ calcd. for $C_{21}H_{36}B_{12}N_8O_5Na$ $[M+Na]^-$: 633.3914; found 633.3914; FT-IR (KBr): 3557, 3365, 2923, 2853, 2484, 2365, 1653, 1508, 1452, 1335, 1160, 1110, 1055, 943, 836, 820, 768 cm^{-1}; mp > 300 °C.

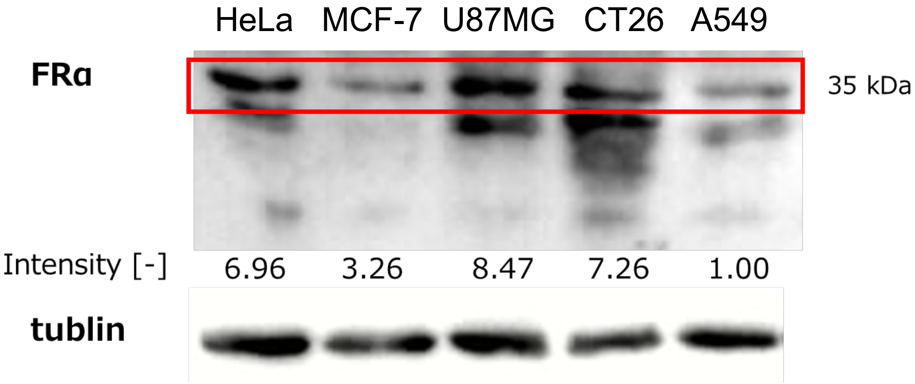

Figure S1. Western blot analysis of FRα in various cell lines. HeLa cell: human cervical carcinoma; MCF-7 cell: human caucasian breast adenocarcinoma; U87MG: human glioblastoma; CT26 cell: mouse colon cancer; A549: human alveolar adenocarcinoma. Overexpression of FRα was observed in HeLa, U87MG, and CT26 cells. 1×10^6 cells of HeLa, MCF-7, U-87 MG, CT26 or A549 was added 1 mL 5× SDS-PAGE sample buffer, and the mixture was boiled at 95 °C for 5 min and was then subjected to SDS-polyacrylamide gel (10 % acrylamide) electrophoresis (PAGE). Acrylamide gel which obtained by SDS-PAGE was transferred toward membrane filter at the condition of 300 mA for 13 min. Soaked and shaked in immunoblock solution for 1 h. It was washed by TBS (Tris Buffered Saline) for 10 min in 3 times. Primary antibody was added and incubated at the condition of 4 °C for 12 h. HRP-conjugated secondary antibody response at the condition of r.t. for 1 h after washed by TTBS buffer for 10 min in 3 times. The chemi luminescence intensity was measured with imager after added detection reagent.

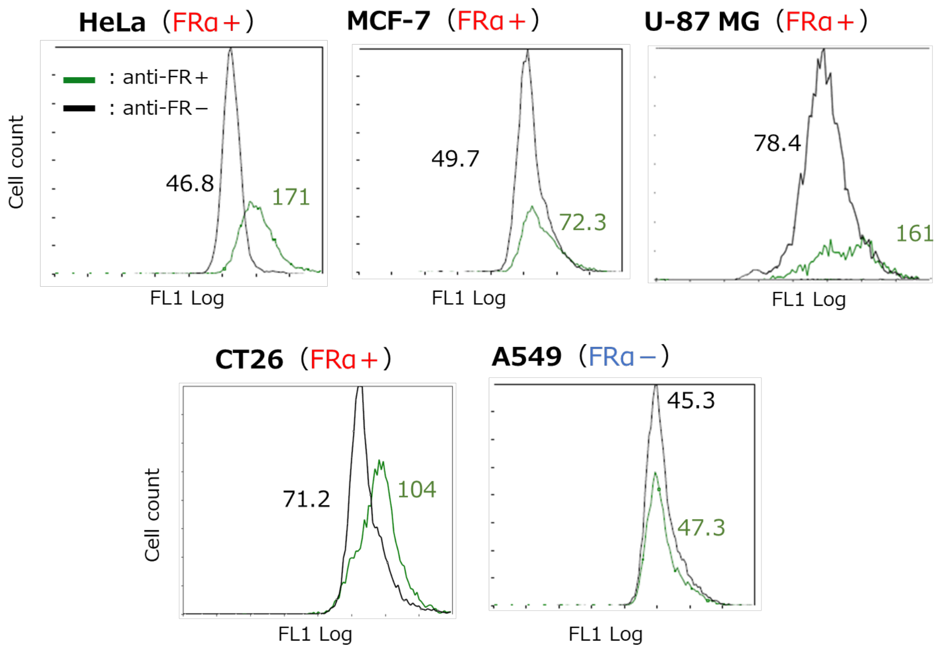

Figure S2. Quantitative analysis of folate receptor (FRα) expressed in HeLa, MCF-7, U87MG, CT26, and A549 cells by flow cytometry using anti-FRα antibody. 5×10^5 cells of HeLa, MCF-7, U-87 MG, CT26 or A549 were washed with 1 mL PBS and then added immunoblock solution. After incubating it for 15 min at 4 °C, the solution was centrifuged (3 min, 2000 rpm). Then supernatant was removed and primary antibody (20 μg/mL in PBS) was added. After incubating it for 30min at 4°C, the solution was centrifuged and the supernatant was removed. After washed with PBS in three times, FITC-conjugated secondary antibody (2 μg/mL in PBS) was added. After incubating it for 30min at 4°C, the solution was centrifuged and the supernatant was removed and washed with PBS in three times. And this cell pellet was dissolved with 500 μL PBS. Fluorescence signals were observed using a flow cytometer.

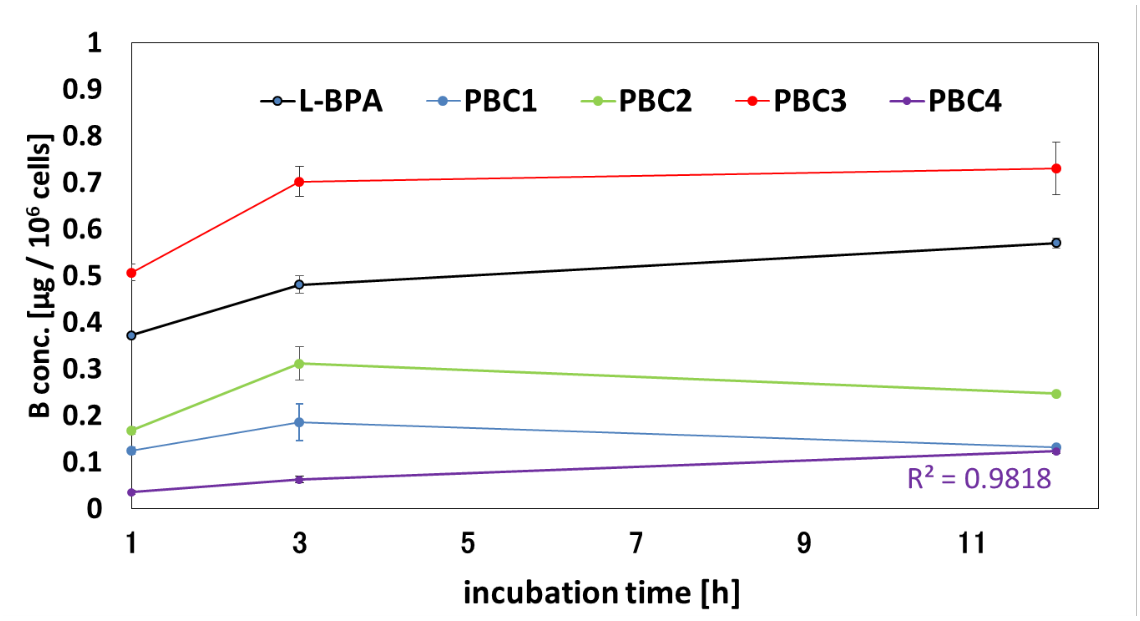

Figure S3. Time-dependent boron accumulation in HeLa cells. The cells (1 x 10^6 cells) were incubated at 37 ˚C for 3 h in a medium containing boron compounds (100 ppm [B]). After the incubation, the cells were washed three times with PBS and digested with 2 mL of perchloric acid/hydrogen peroxide at 70 ˚C 1 h. Boron concentrations in the solution were determined by an inductively coupled plasma optical emission spectroscopy (ICP-OES).

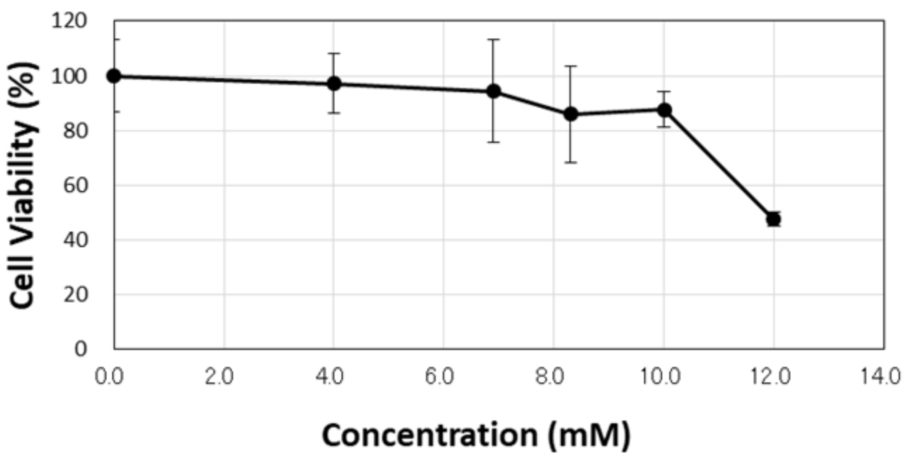

Figure S4. The concentration-dependent cell viability of folate in HeLa cells. Significant antiproliferative effect was not observed up to a 12 mM concentration of folate.
